# Simvastatin attenuates ventilator-induced lung injury in mice

**DOI:** 10.1186/cc9209

**Published:** 2010-07-30

**Authors:** Holger C Müller, Katharina Hellwig, Simone Rosseau, Thomas Tschernig, Andreas Schmiedl, Birgitt Gutbier, Bernd Schmeck, Stefan Hippenstiel, Harm Peters, Lars Morawietz, Norbert Suttorp, Martin Witzenrath

**Affiliations:** 1 Department of Infectious Diseases and Pulmonary Medicine, Charité - Universitätsmedizin Berlin, Charitéplatz 1, 10117 Berlin, Germany; 2 Institute for Anatomy and Cell Biology, Saarland University Faculty of Medicine, Kirrberger Straße, Building 61, 66421 Homburg Saar, Germany; 3 Institute for Functional and Applied Anatomy, Medical School of Hannover, Carl-Neuberg-Str. 1, 30625 Hannover, Germany; 4 BMBF-Forsys Junior Research Group "Systems Biology of Lung Inflammation (FORSYS Lung)", Charitéplatz 1, 10117 Berlin, Germany; 5 Department of Nephrology, Charité - Universitätsmedizin Berlin, Charitéplatz 1, 10117 Berlin, Germany; 6 Institute of Pathology, Charité - Universitätsmedizin Berlin, Charitéplatz 1, 10117 Berlin, Germany

## Abstract

**Introduction:**

Mechanical ventilation (MV) is a life saving intervention in acute respiratory failure without alternative. However, particularly in pre-injured lungs, even protective ventilation strategies may evoke ventilator-induced lung injury (VILI), which is characterized by pulmonary inflammation and vascular leakage. Adjuvant pharmacologic strategies in addition to lung protective ventilation to attenuate VILI are lacking. Simvastatin exhibited anti-inflammatory and endothelial barrier stabilizing properties *in vitro *and *in vivo*.

**Methods:**

Mice were ventilated (12 ml/kg; six hours) and subjected to simvastatin (20 mg/kg) or sham treatment. Pulmonary microvascular leakage, oxygenation, pulmonary and systemic neutrophil and monocyte counts and cytokine release in lung and blood plasma were assessed. Further, lung tissue was analyzed by electron microscopy.

**Results:**

Mechanical ventilation induced VILI, displayed by increased pulmonary microvascular leakage and endothelial injury, pulmonary recruitment of neutrophils and Gr-1^high ^monocytes, and by liberation of inflammatory cytokines in the lungs. Further, VILI associated systemic inflammation characterized by blood leukocytosis and elevated plasma cytokines was observed. Simvastatin treatment limited pulmonary endothelial injury, attenuated pulmonary hyperpermeability, prevented the recruitment of leukocytes to the lung, reduced pulmonary cytokine levels and improved oxygenation in mechanically ventilated mice.

**Conclusions:**

High-dose simvastatin attenuated VILI in mice by reducing MV-induced pulmonary inflammation and hyperpermeability.

## Introduction

In acute respiratory failure, mechanical ventilation (MV) is a life saving treatment without alternatives, and MV is also employed following surgery or trauma. One third of all patients in intensive care units worldwide receive MV [1]. However, particularly in preinjured lungs even minimal MV-associated physical stress may evoke ventilator-induced lung injury (VILI), an important undesirable effect of respirator therapy [2,3]. VILI is characterized by a pulmonary inflammatory response with the liberation of cytokines, recruitment of leukocytes to the lung and increased lung permeability, consecutively resulting in lung edema, surfactant dysfunction, impaired lung compliance and deterioration of pulmonary gas exchange [4]. Clinical studies of Amato *et al. *and the ARDS Network revealed that minimization of MV-induced physical stress by reduction of tidal volumes to 6 ml/kg significantly improved the clinical outcome of mechanically ventilated patients [5,6]. However, even low tidal volume ventilation of healthy lungs causes lung injury [7], and particularly preinjured lungs are sensitive to the development of VILI even in the setting of lung-protective ventilation [2,3]. As the necessity to guarantee sufficient gas exchange limits a further substantial reduction of tidal volumes, new adjuvant pharmacological therapies in addition to lung-protective ventilation are needed to prevent VILI.

Simvastatin, a 3-hydroxy-3-methylglutaryl coenzyme A (HMG-CoA) reductase inhibitor belonging to the group of statins may be a promising drug candidate for adjuvant pharmacotherapy in MV. Besides well-known lipid lowering properties, simvastatin exhibits pleiotropic effects that attenuated acute lung injury (ALI), including reduction of pulmonary microvascular leakage, limitation of pulmonary leukocyte infiltration, and attenuation of pulmonary and systemic hyperinflammation in different experimental settings [8-11]. Moreover, statins may alter inflammatory responses in humans. Healthy volunteers subjected to lipopolysacharide (LPS) inhalation developed lung inflammation, which was attenuated by simvastatin treatment [12]. Further, statin treatment was associated with improved survival in sepsis and severe community acquired pneumonia [13-16].

Pulmonary and systemic hyperinflammation, leukocyte recruitment to the lungs, and the development of pulmonary microvascular leakage are crucial components of VILI [4,17]. We thus hypothesized that simvastatin may reduce VILI and may be a promising adjuvant pharmacologic strategy to limit VILI in addition to lung protective ventilation.

In the current study, anesthetized mice were subjected to mechanical ventilation for six hours. Simvastatin treatment markedly attenuated ventilator-induced pulmonary microvascular permeability and endothelial injury, recruitment of neutrophils and Gr-1^high ^monocytes, as well as proinflammatory cytokine levels in the lung, and improved oxygenation considerably.

## Materials and methods

### Mice

Female C57BL/6 mice (11 to 15 weeks, 20 to 22 g) (Charles River, Sulzfeld, Germany) were employed. Procedures were approved by institutional and governmental authorities.

### Mechanical ventilation

Mice were anesthetized by intraperitoneal injections of Fentanyl (0.075 mg/kg), Midazolam (1.5 mg/kg) and Medetomedin (0.75 mg/kg). Repetitive applications of Fentanyl (0.016 mg/kg), Midazolam (0.33 mg/kg) and Medetomedin (0.16 mg/kg) were done via an intraperitoneal catheter when required to guarantee adequate anaesthesia over the whole experiment. Body-temperature was maintained at 37°C by a heating pad. After tracheotomy and intubation, mice were ventilated (MiniVent, Hugo-Sachs-Electronics, March-Hugstetten, Germany) with 50% oxygen; tidal volume (V_T_) 7 ml/kg; respiratory rate (RR) 240 minute^-1^; positive end-expiratory pressure (PEEP) 6 cmH_2_O. A carotid artery catheter was placed for blood pressure monitoring and infusion of NaCl 0.9% containing 100 mmol/l HCO_3_^- ^(350 μl/h). There was no additional fluid support in any conducted experiment. A urinary catheter was inserted. V_T_, RR, airway pressure, peripheral oxygen saturation and urine output were monitored (Pulmodyn, Hugo-Sachs-Electronics, March-Hugstetten, Germany; MouseOx, STARR Life-Sciences, Oakmont, PA, USA). After preparation, a recruitment maneuver was performed (airway pressure 35 cmH_2_O for 5 sec) before respirator settings were adjusted for 6 h to V_T _12 ml/kg, RR 120 minute^-1^, PEEP 2 cmH_2_O. All mice survived the protocol. At termination of the experiments mice were sacrificed by exsanguination via the carotid catheter. Non-ventilated mice served as controls.

### Simvastatin treatment

Simvastatin (Sigma, Steinheim, Germany) was dissolved in ethanol and diluted with saline. Mice received i.p. injections of 20 mg/kg simvastatin or solvent 24 h and 1 h before the VILI experiment. Non-ventilated mice were treated in according intervals. Simvastatin treatment had no impact on overall cholesterol, HDL and LDL cholesterol in studied mice.

### Blood gas analyses

Blood samples were analyzed for p_a_O_2_, p_a_CO_2_, ph, HCO_3_^-^, SBE, Lactate, Na^+^, K^+^, Cl^-^, Ca^2+ ^by blood gas analyzer (ABL-800, Radiometer, Copenhagen, Denmark). P/F ratio was calculated as p_a_O_2 _/FiO_2_.

### Lung permeability

Human-Serum-Albumin (HSA; 1 mg) was injected via carotid artery catheter or tail vein in ventilated or non-ventilated mice, respectively, 90 minutes before the experiment termination. Mice were sacrificed and bronchoalveolar lavage (BAL) of the right lung was performed with 2 × 400 μl saline. BAL- and plasma HSA-levels were quantified by ELISA (enzyme-linked immuno sorbent assay) (Bethyl (biomol), Hamburg, Germany). Permeability was assessed by calculating the HSA BAL/plasma ratio.

### Electron microscopy

Lungs were flushed via the pulmonary artery, cut, immersion-fixed (1.5% glutaraldehyde, 1.5% paraformaldehyde in 0.15 M HEPES), rinsed (0.1 mmol/l HEPES, 0.1 mmol/l cacodylate buffer) and osmicated (1% osmium tetroxide in 0.1 mmol/l cacodylate buffer). After rinsing in 0.1 mmol/l cacodylate buffer and distilled water, specimens were stained in half-saturated aqueous uranylacetate solution (1:1). Samples were dehydrated in ascending acetone concentrations, embedded in epon, cut (70 nm), stained with lead citrate and uranyl-acetate, and analyzed.

### Differential cell count lung

Lungs were flushed. The left lung was digested in RPMI containing Collagenase and DNAse for 1 h. Leukocytes were extracted by meshing the lung tissue through a cell strainer (100 μm) and counted by haemocytometer and differentiated by flowcytometry according to their side-scatter/forward-scatter properties and CD45, Gr-1 and F4-80 expression.

### Differential cell count blood

Leukocytes were quantified by flowcytometry using TruCount-Tubes and differentiated according to their side-scatter/forward-scatter properties and CD45 and Gr-1 expression.

### Quantification of cytokines

Cytokines were quantified from total protein of flushed homogenized left lungs and blood samples (BioRad, Hercules, CA, USA).

### Measurement of Alanine transaminase levels

Alanine transaminase (ALT) levels were measured by routine laboratory test at the Institute of Laboratory Medicine and Pathobiochemistry of the Charité - Universitätsmedizin Berlin.

### Statistic analyses

Groups were compared using One-Way-ANOVA following Newman-Keuls post test. For comparison of two groups Mann-Whitney U-Test was applied. *P-*values < 0.05 were considered significant. Data are represented as mean +/- SEM.

## Results

### Simvastatin prevented oxygenation failure in VILI

**Figure 1:**
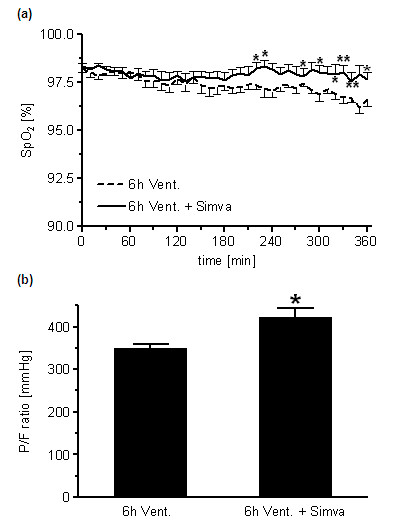
**Simvastatin improved oxygenation in VILI**. **(a) **Peripheral Oxygen Saturation (SpO_2_) was monitored continuously and **(b) **P/F ratio was assessed at the end of the 6 h ventilation period in simvastatin (6 h Vent. + Simva) or sham (6 h Vent.) treated mice. Simvastatin treatment prevented the decline of SpO_2 _and improved oxygenation in VILI. (a: 6 h Vent. *N *= 8; 6 h Vent. + Simva *n *= 10; b: 6 h Vent. *N *= 7; 6 h Vent. + Simva *n *= 9; *P < 0.05).

The decline of the peripheral oxygen saturation (SpO_2_) observed in ventilated mice was prevented by Simvastatin treatment (Figure 1a). At the termination of the experiment, blood gas analysis was performed in arterial blood samples. The P/F ratio was higher in simvastatin treated mice (Figure 1b).

### Simvastatin reduced VILI-associated pulmonary vascular leakage

**Figure 2:**
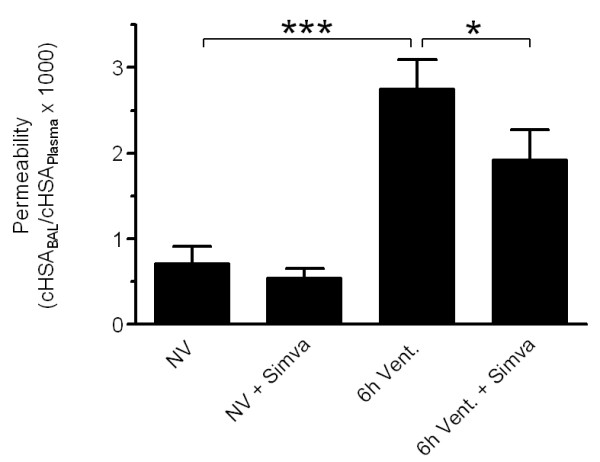
**Simvastatin reduced VILI-associated lung hyperpermeability**. Human serum albumin (HSA; 1 mg) was injected 90 minutes prior to termination of the experiment. In non-ventilated simvastatin (NV + Simva) or sham (NV) treated mice, and in ventilated and simvastatin (6 h Vent. + Simva) or sham (6 h Vent.) treated mice, HSA levels in plasma and BAL were determined. Simvastatin treatment reduced VILI associated lung hyperpermeability. (NV *n *= 6; NV + Simva *n *= 7; 6 h Vent. *N *= 7; 6 h Vent. + Simva *n *= 6; *P < 0.05, ***P < 0.001).

MV induced a marked increase of pulmonary microvascular permeability in mice, indicated by an elevated HSA BAL/plasma ratio. Pulmonary hyperpermeability was decreased by Simvastatin treatment (Figure 2).

### Simvastatin attenuated endothelial injury in ventilated mice

**Figure 3:**
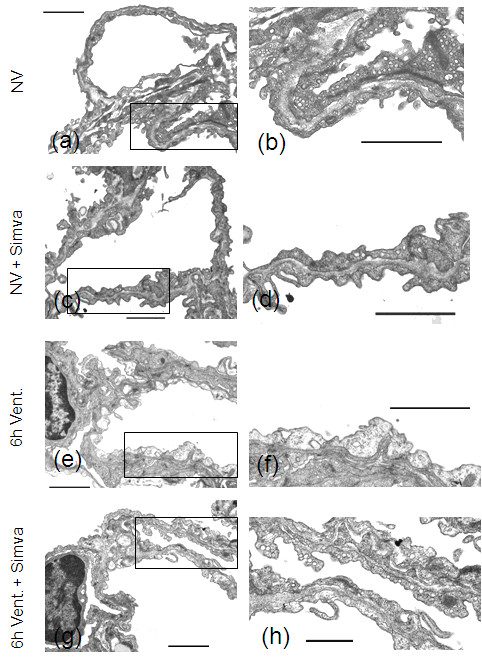
**Simvastatin reduced VILI-associated endothelial injury**. In lung sections of non ventilated, sham treated mice (NV) **(a, b) **and non-ventilated, simvastatin treated mice (NV + Simva) **(c, d)**, structurally intact capillaries containing numerous caveolae and vesicles in endothelial cells were seen. In lung sections of ventilated, sham treated mice (6 h Vent.) **(e, f)**, capillaries showed pronounced endothelial cell swelling as well as loss of intracellular vesicles and caveolae. Lungs of ventilated, simvastatin treated mice (6 h Vent. + Simva) **(g, h) **had intact capillaries, and neither signs of endothelial cell swelling, nor reduction of intracellular vesicles was observed. (Representative images out of *n *= 8 each group are shown. Bar 2 μm).

Non-ventilated, untreated or simvastatin treated mice exhibited intact alveolar epithelium and capillary endothelium (Figure 3a-d). Capillary endothelial cells of ventilated and untreated mice were swollen and showed loss of intracellular vesicles and caveolae (Figure 3e, f). In ventilated and simvastatin treated lungs, endothelial cells displayed fewer signs of injury as compared to ventilated and untreated mice. Swelling of endothelial cells occurred only sporadically, and normal distribution of vesicles and caveolae was preserved by simvastatin (Figure 3g, h).

### Simvastatin limited the recruitment of PMN and Gr-1^high ^monocytes to the lung in VILI

**Figure 4:**
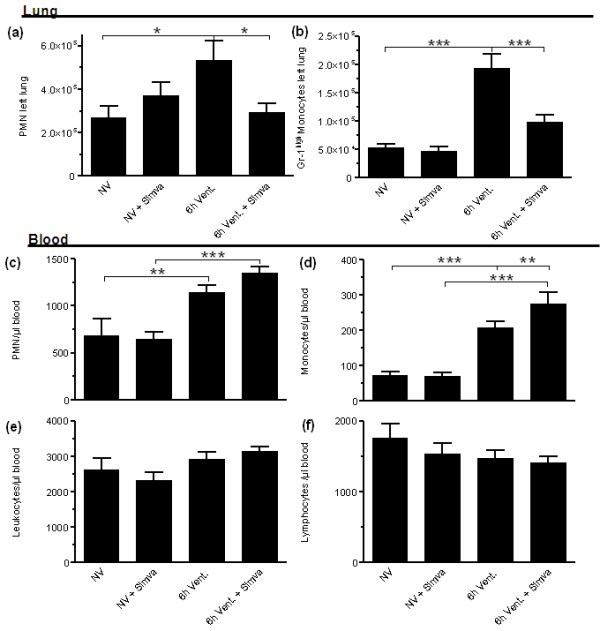
**Simvastatin treatment limited VILI-associated pulmonary leukocyte infiltration**. After 6 h mechanical ventilation (MV) of simvastatin (6 h Vent + Simva) or sham treated mice (6 h Vent.) and in non-ventilated sham (NV) or simvastatin (NV + Simva) treated mice, leukocytes isolated from whole left lung tissue and from blood were differentiated by flow cytometry. MV increased pulmonary PMN **(a) **and GR-1^high ^monocytes **(b)**. Simvastatin reduced PMN and monocyte counts in the lungs of ventilated mice. MV also increased circulating blood neutrophils **(c) **and Gr-1^high ^monocytes **(d)**, whereas leukocyte **(e) **and lymphocyte **(f) **counts were not significantly altered by MV (F). PMN and Gr-1^high ^monocyte counts were higher in Simvastatin treated, ventilated mice (6 h Vent. + Simva), as compared to sham treated, ventilated mice (6 h Vent.). (a-b: NV *n *= 6; NV + Simva *n *= 7; 6 h Vent. *N *= 7; 6 h Vent. + Simva *n *= 6. c-d: NV *n *= 9; NV + Simva *n *= 9; 6 h Vent. *N *= 8; 6 h Vent. + Simva *n *= 8; *P < 0.05; **P < 0.01, ***P < 0.001).

MV evoked PMN and Gr-1^high ^monocyte recruitment to the lung, which was reduced by simvastatin treatment (Figure 4a, b). Further, MV elicited an increase of circulating PMN and monocyte counts, whereas lymphocyte counts were unaltered in the blood (Figure 4c-f). Notably, following simvastatin treatment monocyte counts were increased significantly and PMN counts were increased by trend in blood of ventilated mice (Figure 4c, d).

### Simvastatin treatment attenuated VILI-associated pulmonary cytokine production

**Figure 5:**
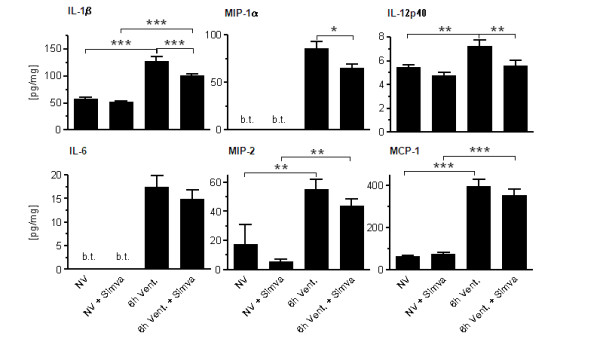
**Simvastatin attenuated VILI-associated pulmonary cytokine production**. Simvastatin (6 h Vent. + Simva) or sham treated mice (6 h Vent.) subjected to 6 h of mechanical ventilation, and non-ventilated sham (NV) or simvastatin (NV + Simva) treated mice were sacrificed. Cytokine levels in homogenized lung tissue were quantified. MV evoked an increase of pulmonary cytokines. Simvastatin treatment limited VILI associated production of IL-1β, MIP-1α and IL-12p40. (*n *= 8 each group; *P < 0.05, **P < 0.01, ***P < 0.001). (b.t., below threshold).

MV induced an increase of IL-1β, IL-6, IL-12p40, MIP-1α, MIP-2 and MCP-1 in the lung tissue. Simvastatin treatment attenuated the ventilation-evoked increase of IL-1β, IL-12p40 and MIP-1α in the lung tissue (Figure 5).

### Simvastatin treatment attenuated VILI-associated IL-12p40 increase in plasma

MV evoked an increase of IL-1β, IL-6, IL-12p40, MIP-1α, MIP-2 and MCP-1 in blood plasma. Simvastatin treatment attenuated the VILI-associated increase of IL-12p40 in the plasma. All other quantified cytokines did not show statistically significant alterations due to simvastatin treatment in ventilated mice (Table 1).

**Table 1:** Simvastatin treatment reduced IL-12p40 levels in plasma

	NV		NV + Simva		**6 h Vent**.		6 h Vent. + Simva	
				
	mean (pg/μl)	SD	mean (pg/μl)	SD	mean (pg/μl)	SD	mean (pg/μl)	SD
**IL-1β**	445.30	100.70	505.30	52.19	763.2 ***	95.38	738.8 ###	100.40
**IL-6**	57.58	62.99	133.40	90.38	915.3 ***	459.00	993.9 ###	364.60
**IL-12p40**	462.10	99.32	593.10	128.60	2,352 ***	783.40	1,785 ###, ^a^	372.50
**MIP-1α**	307.20	149.10	386.50	78.94	918.6 ***	318.60	900.8 ###	128.20
**MIP-2**	15.28	35.02	10.03	9.42	95.32 **	49.21	67.25 #	23.73
**MCP-1**	99.84	17.33	153.50	29.41	758.9 *	404.90	559.70	133.30

Cytokine levels were assessed in blood plasma after 6 h mechanical ventilation (MV) of simvastatin (6 h Vent + Simva) or sham treated mice (6 h Vent.) and in plasma of non-ventilated simvastatin (NV + Simva) or sham (NV) treated mice. MV induced a systemic inflammatory response indicated by elevated cytokine levels in blood. Simvastatin treatment reduced IL-12p40 levels in plasma significantly. The levels of IL-1β, MIP-1α, IL-6, MIP-2 and MCP-1 in VILI did not show statistically significant alterations due to simvastatin treatment. (*n *= 8 each group; * *P *< 0.05, ** *P *< 0.01, *** *P *< 0.001 vs. NV; # *P *< 0.05, ### *P *< 0.001 vs. NV + Simva; ^a ^*P *< 0.05 vs. 6 h Vent.).

### Hemodynamics, urine output electrolytes, acid-base homeostasis and markers of hepatic and renal function

Continuous monitoring of systemic arterial blood pressure and quantification of electrolytes, parameters of acid-base homeostasis, renal and global hepatic function and urine output at the end of the experiment demonstrated standardization of experimental procedures.

Simvastatin treatment did not alter blood pressure, urine output electrolyte levels or acid-base homeostasis in mechanically ventilated mice. Further simvastatin had no impact on renal function or ALT levels in plasma (Table 2).

**Table 2:** Hemodynamics, electrolytes, respiratory parameters lactate, ALT and Cystatin C levels and urine output of mechanically ventilated mice

	**6 h Vent**.		6 h Vent. + Simva	
		
	mean	SD	mean	SD
**Na**^**+ **^**(mmol/l)**	159	5	159	4
**K**^**+ **^**(mmol/l)**	4.7	0.5	4.7	0.4
**Cl**^**- **^**(mmol/l)**	124	4	125	4
**Ca**^**2+ **^**(mmol/l)**	1.29	0.06	1.30	0.05

**ph**	7.45	0.08	7.51	0.08
**p**_**a**_**CO**_**2 **_**(mmHg)**	42.2	4.8	34.5	10.0
**SBE (mmol/l)**	3.0	2.1	3.5	1.7
**HCO**_**3**_^**- **^**(mmol/l)**	26.7	2.6	26.6	2.7
**Lactate (mmol/l)**	1.5	0.4	1.6	0.3

**ALT (U/l)**	30	12	21	4
**Cystatin C (ng/ml)**	443	64	479	65

**MAP 0 h (mmHg)**	91	7	87	10
**MAP 1 h (mmHg)**	74	7	72	7
**MAP 2 h (mmHg)**	68	5	70	6
**MAP 3 h (mmHg)**	70	6	70	7
**MAP 4 h (mmHg)**	68	5	69	7
**MAP 5 h (mmHg)**	69	6	67	7
**MAP 6 h (mmHg)**	73	8	72	10

**Urine Output 6 h (μl)**	908	294	669	207

After six hours of mechanical ventilation of simvastatin (6 h Vent. + Simva) or sham treated mice (6 h Vent.) urine output was assed and electrolyte, blood gas, lactate, Cystatin C and ALT analysis was performed from arterial blood or plasma respectively. Systemic mean arterial blood pressure (MAP) was measured continuously.

## Discussion

Mechanical ventilation may evoke ventilator-induced lung injury even under employment of protective ventilation strategies. Adjuvant pharmacologic approaches to reduce VILI in addition to protective ventilation may further improve morbidity and mortality of ventilated patients. Investigating VILI in a mouse model of MV, the current study for the first time provides experimental evidence that simvastatin treatment may limit VILI *in vivo*. Simvastatin reduced VILI-associated hyperpermeability, endothelial injury, neutrophil and monocyte recruitment, and inflammation in murine lungs.

Mouse models have been successfully used to investigate pathomechanisms of VILI [18-20]. The currently employed mouse model allowed us to analyze key features of VILI while avoiding detrimental lung injury due to high airway pressures, tidal volumes or respiration rates. Although a V_T _of 6 ml/kg is recommended for lung protective ventilation, we employed a V_T _of 12 ml/kg which allowed for limitation of respiratory rates in our model, an important independent trigger of VILI in mice [21]. Further lung stress and lung strain, generated by a V_T _of 12 ml/kg affecting healthy lungs in the current model may apply in ventilated areas of inhomogeneously injured lungs even under lung protective ventilation according to the baby lung concept of the inhomogeneous ARDS lung [22,23]. To further enhance clinical relevance, we prevented hemodynamic instability by fluid support and metabolic acidosis by adequate infusion of sodium bicarbonate. In summary, a mouse model was established for the current study, which evoked moderate lung injury by ventilation for a six-hour period.

Microvascular leakage, a hallmark of VILI evokes lung edema, reduction of lung compliance, surfactant dysfunction, and finally deterioration of pulmonary gas exchange [4]. Statins prevented pulmonary hyperpermeability in ALI evoked by different stimuli, including endotoxin and ischemia/reperfusion [8-10]. Of note, simvastatin treatment also reduced VILI-associated pulmonary hyperpermeability and improved pulmonary gas exchange in the current study.

Different mechanisms of endothelial barrier protection by HMG-CoA reductase inhibitors have been reported, including inhibition of the RhoA/Rho kinase pathway with consecutive reduction of endothelial myosin light chain phosphorylation [24-26], stabilization of endothelial junctions by polymerization of cortical actin [25], as well as downregulation of endothelial caldesmon and upregulation of integrin β4 expression in endothelial cells [25]. Although these mechanisms were not evaluated in detail in the current study, they may have been contributing to the observed improvement of barrier function in murine VILI. Notably, an additional way of endothelial cell protection by simvastatin has now been observed by electron microscopy. Simvastatin attenuated VILI-evoked cell swelling and loss of intracellular vesicle structures in lung endothelium, which are indicators of energy depletion and impaired cell metabolism. Previous *in vitro *and *in vivo *studies linked cyclic stretch with apoptosis and necrosis of pulmonary *epithelial *cells [27,28]. In line with the works of Vaneker *et al. *this study provides ultrastructural *in vivo *evidence for lung endothelial cell injury following ventilation with moderate tidal volumes [29]. The observed morphologic findings resemble alterations observed in capillary stress failure previously described by West *et al. *To the best of our knowledge this is the first study showing that a pharmacologic treatment attenuated endothelial injury VILI. This previously undescribed effect of simvastatin treatment suggests a so far unknown beneficial effect of HMG-CoA reductase inhibitors, which may be further examined in future studies.

In VILI, PMN and Gr-1^high ^monocytes infiltrate the lungs and have been identified as major effector cells for the development of tissue damage [30-32]. Reportedly, simvastatin inhibited tissue leukocyte infiltration in ALI both in animal experiments and in humans [8,9,12]. Leukocyte rolling, adhesion and transmigration were attenuated by simvastatin, at least partly by reduction of adhesion molecules including CEACAM-1, VCAM-1 and PCAM-1 [33-36]. In line, the significant recruitment of PMN and Gr-1^high ^monocytes in murine VILI was diminished by simvastatin in the current study. Moreover, an MV-induced increase of circulating PMN and Gr-1^high ^monocytes in the blood was even more pronounced in simvastatin-treated mice. This observation may suggest that simvastatin-evoked inhibition of endothelial leukocyte recruitment contributed to reduced pulmonary and concomitantly increased blood counts of PMN and Gr-1^high ^monocytes.

Simvastatin reduced production and liberation of various cytokines in animal models of ALI, sepsis and asthma as well as in humans following LPS-inhalation [9,11,12,37-40]. In the current study, VILI-associated pulmonary production of IL-1β, MIP-1α and IL-12p40 was reduced by simvastatin treatment. Thus, alteration of chemotaxis may have been contributing to the limitation of PMN and Gr-1^high ^monocyte influx into the lungs in this study. Particularly IL-1β may be a key mediator in VILI, as IL-1β blockade as well as IL-1β deficiency resulted in reduced pulmonary PMN recruitment and hyperpermeability in animal models of VILI [41]. Therefore, dampening of pulmonary IL-1β production by simvastatin may have been adding to the observed attenuation of microvascular leakage, pulmonary leukocyte recruitment and endothelial cell injury.

Although increasing evidence derived from experimental and observational studies suggests beneficial effects of simvastatin in ALI as well as in pneumonia [8-11,14,16,42], a retrospective study analyzing an ALI patient cohort did not find an outcome improvement by conventional statin treatment [43]. Of note, statin doses of 5 mg/kg/d did not improve experimental ALI [8], whereas higher doses of 10 to 20 mg/kg/d evoked protective effects. Further, previous studies suggested a delay of at least 6 h for the development of barrier-protective effects by simvastatin [24,25]. Thus, mice were pretreated with 20 mg/kg/d simvastatin commencing 24 h before the onset of ventilation in the current study. Although mandatory for this experimental approach, simvastatin pre-treatment does not match the clinical scenario. However, animal studies are limited to hours while ARDS patients often are ventilated for days or even weeks. Taking this long time course in account we believe that simvastatin may deliver its beneficial effects over time when it is given with the initation of MV. Notably, an upcoming randomized controlled NHLBI sponsored trial is going to investigate statin therapy in ALI (NCT00979121). As patients included in this trial will presumably receive respirator therapy, the effects of statins on VILI observed in the current experimental study may possibly contribute to the outcome of the treatment arm.

## Conclusions

This study shows, for the first time, that high-dose simvastatin markedly reduced VILI-associated microvascular leakage and improved pulmonary gas exchange in mechanically ventilated mice. Simvastatin prevented recruitment of PMN and Gr-1^high ^monocytes to the lung, limited pulmonary cytokine production and attenuated endothelial injury in VILI. The data suggest that high-dose simvastatin offers a promising perspective to prevent VILI in addition to lung protective ventilation.

## Key messages

• Simvastatin improved microvascular leakage and improved oxygenation in VILI.

• Simvastatin limited pulmonary hyperinflammation in VILI.

• Simvastatin protected against VILI induced pulmonary endothelial injury.

• Simvastatin offers a promising perspective to limit VILI in addition to lung protective ventilation.

## Abbreviations

ALI: acute lung injury; ALT: Alanine transaminase; BAL: bronchoalveolar lavage; ELISA: enzyme-linked immuno sorbent assay; HMG COA: 3-hydroxy-3-methylglutaryl coenzyme A; HAS: human serum albumin; LPS: lipopolysacharide; MV: mechanical ventilation; PEEP: positive end-expiratory pressure; VILI: ventilator-induced lung injury; V_T_: tidal volume pressure.

## Competing interests

The authors declare that they have no competing interests.

## Authors' contributions

HCM designed, coordinated and supervised all experiments, analysed the data and drafted the manuscript. KH and BG carried out the animal experiments and performed flow cytometry experiments. SR contributed to the design of the experiments and drafted the manuscript. TT and AS performed electron microscopy and were responsible for image analysis. BS and SH carried out multiplex array experiments while HP performed cystatin C analysis and NS participated in drafting the manuscript. MW participated in the study design and drafted the manuscript.
